# Distinct Morphological and Behavioural Alterations in ENU-Induced Heterozygous *Trpc7^K810Stop^* Mutant Mice

**DOI:** 10.3390/genes12111732

**Published:** 2021-10-29

**Authors:** Birgit Rathkolb, Maike Howaldt, Stefan Krebs, Petra Prückl, Susanne Sauer, Martin Hrabě de Angelis, Bernhard Aigner

**Affiliations:** 1 Chair for Molecular Animal Breeding and Biotechnology, Laboratory for Functional Genome Analysis (LAFUGA), Gene Center, LMU Munich, 81377 Munich, Germany; birgit.rathkolb@helmholtz-muenchen.de (B.R.); m_howaldt@yahoo.de (M.H.); krebs@genzentrum.lmu.de (S.K.); petra.prueckl@gmx.net (P.P.); 2 German Mouse Clinic, Institute of Experimental Genetics, Helmholtz Zentrum München, 85764 Neuherberg, Germany; hrabe@helmholtz-muenchen.de; 3 German Center for Diabetes Research (DZD), 85764 Neuherberg, Germany; 4 Institute of Physiology and Pathophysiology, Friedrich-Alexander University Erlangen Nürnberg, 91045 Erlangen, Germany; susanne.sauer@fau.de; 5 Chair of Experimental Genetics, School of Life Science Weihenstephan, Technische Universität München, 85354 Freising, Germany

**Keywords:** animal model, growth, seizure, tissue irritation, TRPC7

## Abstract

*Trpc7* (transient receptor potential cation channel, subfamily C, member 7; 862 amino acids) knockout mice are described showing no clear phenotypic alterations, therefore, the functional relevance of the gene remains unclear. A complementary approach for the functional analysis of a given gene is the examination of individuals harbouring a mutant allele of the gene. In the phenotype-driven Munich ENU mouse mutagenesis project, a high number of phenotypic parameters was used for establishing novel mouse models on the genetic background of C3H inbred mice. The phenotypically dominant mutant line SMA002 was established and further examined. Analysis of the causative mutation as well as the phenotypic characterization of the mutant line were carried out. The causative mutation was detected in the gene *Trpc7* which leads to the production of a truncated protein due to the novel stop codon at amino acid position 810 thereby affecting the highly conserved cytoplasmic C terminus of the protein. *Trpc7* heterozygous mutant mice of both sexes were viable and fertile, but showed distinct morphological and behavioural alterations which is in contrast to the published phenotype of *Trpc7* knockout mice. Thus, the *Trpc7^K810Stop^* mutation leads to a dominant negative effect of the mutant protein.

## 1. Introduction

*Trpc7* (transient receptor potential cation channel, subfamily C, member 7) encodes a receptor-activated non-selective calcium permeable cation channel which is expressed in neurons and glia in the brain as well as in other tissues. The murine *Trpc7* gene comprises 12 exons and encodes a 862 amino acid protein consisting of a N terminal cytoplasmic domain, six helical transmembrane domains with the pore region located between transmembrane domains 5 and 6, and the cytoplasmic C terminus (aa 673-862). The amino acid sequence of the protein is highly conserved among different species including the C terminal cytoplasmic domain (http://www.ensembl.org; http://www.uniprot.org, accessed on 31 August 2021). Among the mammalian TRPC family consisting of seven members (TRPC1-TRPC7), TRPC7 shows a high degree of amino acid sequence homology to and selectively interacts with TRPC3 and TRPC6. These three members of the TRPC family belong to a subgroup that can be directly activated by diacylglycerol (DAG) [1,2].

The MGI database (http://www.informatics.jax.org) harbouring knockout as well as mutant mouse alleles includes three published *Trpc7* mouse mutants (accessed on 31 August 2021). The chemically induced line In(13)31Rk exhibits a large inversion on MMU13 including *Trpc7*, and the heterozygous mutant males of this line showed abnormal meiosis. The second line listed (*Trpc7*^tm1.1Clph^) exhibits the targeted deletion of exon 1 of *Trpc7* and was used in the analysis of melanopsin signaling in mammalian iris and retina [3]. The same topic was examined by using the third line listed (*Trpc7*^tm1.1Lbi^) which exhibits the targeted deletion of exon 5 of *Trpc7*. Homozygous mutant mice of this line were described to be viable and to show no obvious differences from wild-type littermates in weight and size [4]. In addition, mice lacking expression of all seven members of the TRPC family (TRPC1-TRPC7) were viable [5].

Using the TRPC7 knockout mice in a mixed 129/Sv and C57BL/6 genetic background, the genetic ablation of TRPC7 was shown to disrupt acute severe seizures induced by pilocarpine in mice. This disruption was associated with a reduction in pilocarpine-induced increase in gamma wave activity that precedes the acute seizures [6].

The same TRPC7 knockout mice were used to examine TRPC7 function in the skin. TRPC7 was characterized as a nociceptive mechanoreceptor, and it was described to mediate UVB-induced epidermal pathology, epidermal aging, and skin tumour initiation and growth in mice [7]. In summary, due to the lack of clear un-induced phenotypic alterations in *Trpc7* knockout mice, the functional relevance of the gene remains unclear.

In humans, TRPC7 genetic variants were detected to modify risk for lung cancer [8]. In addition, a role of TRPC7 in the cardiovascular system was proposed [9,10]. Furthermore, both the Online Mendelian Inheritance in Man (OMIM) database and the publically available database of the *Human Gene Mutation Database* *(HGMD)* listed no human disease-causing TRPC7 mutation (31 August 2021).

A strategy for the search of novel disease-related alleles consists in the random chemical mutagenesis of a large number of animals followed by systematic screening for clinically relevant disease phenotypes. The alkylating agent *N*-ethyl-*N*-nitrosourea (ENU) is mutagenic for premeiotic spermatogonial stem cells and allows the production of a large number of randomly mutagenized offspring from treated males. ENU predominantly induces point mutations. In the phenotype-driven Munich ENU mouse mutagenesis project using C3HeB/FeJ (C3H) inbred mice as genetic background, a standardized screening profile of a high number of phenotypic parameters was established for the analysis of offspring of mutagenized mice in order to detect phenotypic variants [11].

The ENU mutagenesis-derived dominant mutant mouse line SMA002 was established showing the combined appearance of growth deficit and abnormal behaviour as mutant phenotype, and was analyzed for the causative mutation. A single base exchange was identified leading to the establishment of the mutant mouse line *Trpc7^K810Stop^*.

## 2. Materials and Methods

### 2.1. Animals and Linkage Analysis of the Causative Mutation

The dominant mutant line SMA002 was established in the phenotype-based Munich ENU mouse mutagenesis project [12] on the C3HeB/FeJ (C3H) inbred genetic background by detecting an abnormal phenotype including both growth deficit as well as abnormal behaviour. Mouse husbandry, breeding, linkage analysis, and genome-wide mapping were performed as described previously [13]. All mice had free access to drinking water and a standard rodent diet (V1124, Ssniff, Soest, Germany; Altromin chow #1314, Altromin, Lage, Germany) ad libitum.

For linkage analysis of the causative mutation, a genome-wide mapping panel consisting of 57 polymorphic microsatellite markers was applied. The markers used are available upon request. Additional fine mapping was performed using further microsatellite and SNP markers. Chromosomal positions of markers and genes are according to the GRCm38.p6 mouse genome assembly, 2019 (http://www.ensembl.org, accessed on 31 August 2021).

### 2.2. Exome Sequencing

Genomic DNA from C3H controls and two mice phenotypically heterozygous for the putative mutation was sheared by sonication (Bioruptor, Diagenode, Liege, Belgium), end-repaired, A-tailed and ligated to Illumina adapters. The resulting whole genome sequencing libraries were amplified by six cycles of PCR and then hybridized to a mouse whole exome bait library. Fragments complementary to the biotinylated exome bait library were enriched by pull-down with paramagnetic streptavidin-coated beads (Dynabeads M280, Invitrogen, Waltham, MA, USA) and finally amplified with barcoded Illumina adapters. All previously described steps used reagents from the Agilent whole exome kit and followed the protocol of the manufacturer. The resulting exome libraries were purified with Ampure XP beads (Beckman-Coulter, Brea, CA, USA), quantified and assessed on the Bioanalyser (Bioanalyser 2100, Agilent, Santa Clara, CA, USA). Pooled, barcoded libraries were sequenced on an Illumina Genome Analyzer IIx in paired-end mode with a read length of 80 bp in either direction. Sequence reads in fastq format were demultiplexed, adapter-clipped and quality filtered. After mapping to the mouse genome with BWA, SNPs were called using VARSCAN. Only SNPs that were exclusively called in the mutant mice and not in any of the three controls were kept for further evaluation.

### 2.3. Phenotypic Analysis

Mouse husbandry was done under a continuously controlled specific pathogen free (SPF) hygiene standard according to the FELASA recommendations [14] (http://www.felasa.eu, accessed on 31 August 2021). All tests were carried out under the approval of the responsible animal welfare authority (Regierung von Oberbayern, Munich, Germany).

Data are shown as mean ± standard deviation. Statistically significant differences are indicated for *p* < 0.05, 0.01, and 0.001.

## 3. Results

### 3.1. Generation and Basal Phenotypic Analysis of Line SMA002

The ENU mutagenesis-derived, dominant mutant line SMA002 with a G1 founder was established on the C3H inbred genetic background. Maintenance of the line involved repeated backcross of phenotypically heterozygous mutant mice to C3H wild-type mice for more than ten generations, leading to the subsequent loss of all non-causative ENU mutations not linked to the mutation causing the line-specific abnormal phenotype. Complete penetrance of the mutant phenotype was observed in offspring of matings of phenotypically heterozygous mutant mice to wild-type C3H mice as expected by the rules of Mendelian inheritance. Heterozygous mutants of both sexes were viable and fertile, however, due to the debilitating mutant phenotype usually male heterozygotes were mated to C3H females for the propagation of the line and line breeding was continued only with few animals to minimize the burden of the animals in the breeding procedure.

**Figure 1:**
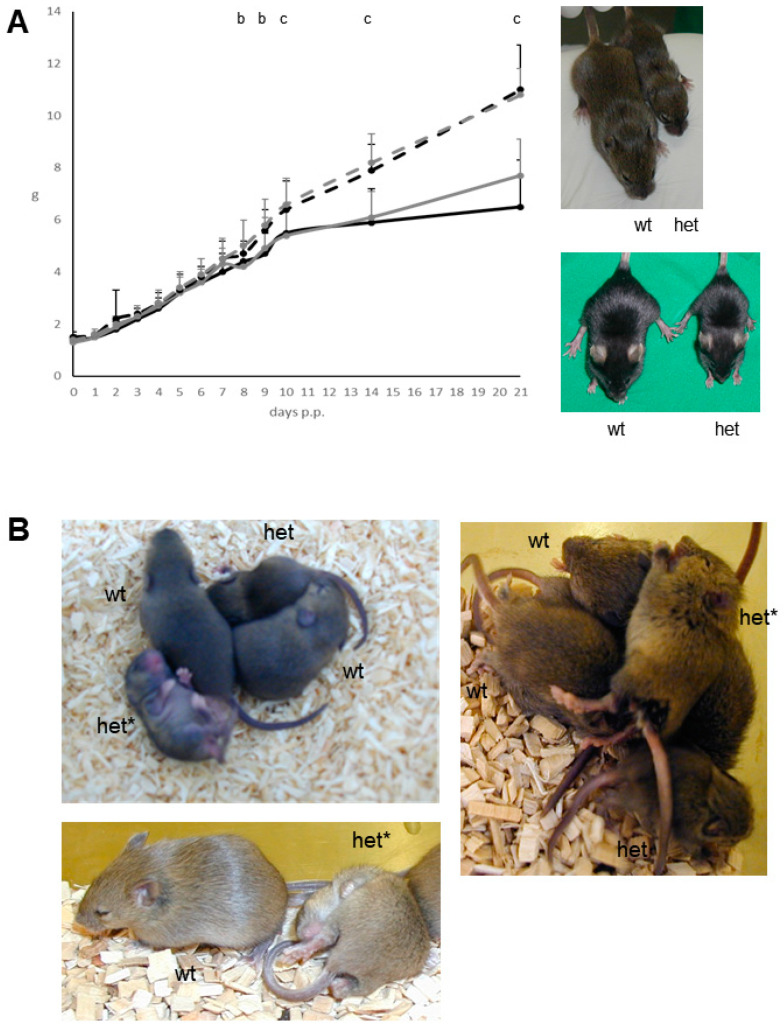
Growth deficit and abnormal behavioural phenotype of mutant line *Trpc7^K810Stop^*. (**A**) Development of body weight (mean ± standard deviation) in heterozygous mutant male (black) and female (grey) mice and C3H littermate controls (dashed lines) during the first three weeks post partum. *n* = 11–34 per sex and genotype. Significance vs. wild-type controls: b, *p* < 0.01 (day 8 p.p. only for females); c, *p* < 0.001. Reduced body weight as well as reduced nose-rump length of a female heterozygous mutant mouse (het) at the age of 15 days post partum compared to a female wild-type (wt) C3H littermate (**right** panel, **top**). The analogous phenotype of the mutation is observed in the C57BL/6 genetic background (**right** panel, **bottom**). (**B**) Representative images showing heterozygous mutant mice (het) at the age of 10–20 days post partum compared to wild-type C3H littermates (wt). The heterozygous mutants depicted with a star (het*) underwent seizure-like attacks involving both the body and the limbs during the image taking. In addition, an increased frequency of a probably irritation-induced self-grooming and scratching behaviour of body and limbs was detected.

The abnormal phenotype of the heterozygous mutant mice consists of the combined appearance of growth deficit (Figure 1A) as well as an abnormal behaviour (Figure 1B). The reduced body weight of the heterozygous mutant male (26.7 ± 1.9 g; mean ± standard deviation) and female (23.3 ± 2.5 g) mice also persisted later in life when compared to C3H littermate controls (37.1 ± 4.0 g in males, and 35.1 ± 4.9 g in females) after 26 weeks post partum (*n* = 24–32 per sex and genotype; significance vs. wild-type controls: *p* < 0.001). At this age, the nose-rump length also was reduced in heterozygous mutants vs. sex-matched littermate controls, whereas analysis of relative organ weights (gastrointestinal tract, heart, kidney, liver, lung, pancreas, spleen, thymus) detected no obvious differences (*n* = 6 per sex and genotype).

Starting from the second week post partum and being highly apparent between day 14–28 post partum, the heterozygous mutants show recurrent, uncontrolled appearing abrupt seizure-like attacks involving both the body and the limbs with a time span of several seconds as well as an increased frequency of a probably irritation-induced self-grooming and scratching behaviour of body and limbs (Figure 1B). Later in life, this abnormal behaviour became less apparent at least during our observations in the normal light phase of the mouse husbandry. Thus, in this stage of life the persistent growth deficit was used as the main mutant phenotype to discriminate wild-type and mutant mice.

After the transfer of the causative mutation to the C57BL/6J genetic background by backcrossing heterozygous mutants to C57BL/6J inbred mice for more than ten generations, the analogous abnormal phenotype of growth deficit and abnormal behaviour was also observed in the heterozygous mutants of this congenic line. In addition, older mutant mice of the congenic line inconstantly showed skin alterations of various degrees including the neck region. It is not clear if this appeared as a consequence of the increased self-grooming and scratching behaviour leading to skin defects.

The growth deficit of the heterozygous mutants was further examined by analyses of serum growth factors in adult male mice (*n* = 6 per genotype). Insulin-like growth factor 1 (IGF1) was decreased (mean ± standard deviation of the heterozygous mutant males: 323 ± 27 ng/mL versus 434 ± 48 ng/mL in controls; *p* < 0.001) and insulin-like growth factor binding protein 2 (IGFBP2) was increased (mean ± standard deviation of the heterozygous mutant males: 685 ± 101 ng/mL versus 551 ± 105 ng/mL in controls; *p* < 0.05) in heterozygous mutants, whereas no difference was found for insulin-like growth factor 2 (IGF2; mean ± standard deviation of the heterozygous mutant males: 28 ± 4 ng/mL versus 27 ± 3 ng/mL controls).

The clinical chemical analysis of blood plasma at the age of twelve weeks included the additional comparison to an external C3H population and revealed significantly decreased values for the parameters potassium, total protein, cholesterol and triglycerides in both sexes of the heterozygous mutants compared to wild-type littermate controls. The hematological analysis observed no obvious differences between heterozygous mutant mice and wild-type littermate controls (Table 1). Thus, the analyses excluded the appearance of systemic liver and/or kidney diseases leading to pruritus with the consequence of scratching skin defects. Additionally, analysis of basal immunology parameters (IgA, IgE, IgG3, IgM, Rf and anti-DNA-ab) in blood plasma as well as the histological analysis of skin biopsies observed no obvious differences between heterozygous mutant mice and wild-type littermate controls. In addition, in vitro investigations on skin tissue response to different nociceptive stimuli revealed no genotype-related differences but a subtle reduction in histamine release in response to environmental acidification.

### 3.2. Identification of the Causative Mutation in the Gene Trpc7

Genome-wide linkage analysis of the causative mutation was carried out with phenotypically heterozygous mutant G2 animals derived from two consecutive backcross matings of phenotypically heterozygous mutants to C57BL/6J inbred mice. Using a set of 57 polymorphic microsatellite markers, the mutant phenotype was mapped on MMU 13 to D13Mit20 (55.6 Mb) and D13mit253 (64.0 Mb). Further fine mapping using 229 phenotypically heterozygous mutant G2 animals showed the highest χ^2^ value for the polymorphic marker D13Mit13 (56.5 Mb; χ^2^ value: 167).

**Figure 2:**
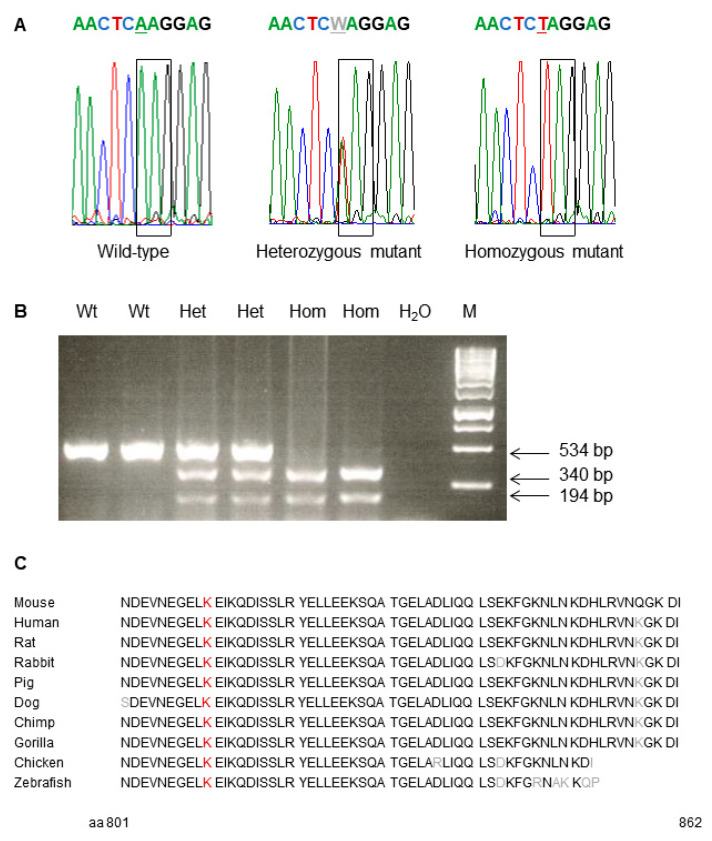
Analysis of the causative point mutation in mutant and wild-type mice of line *Trpc7^K810Stop^*. (**A**) Electropherogram of the causative point mutation. The box shows the AAG → TAG exchange at the amino acid position 810. (**B**) Genotyping of mice by allele-specific PCR-RFLP reaction. FspBI restriction digest of the 534 bp PCR product results in 340 bp and 194 bp fragments of the mutant allele. Hom, *Trpc7^K810Stop^* homozygous mutant; Het, *Trpc7^K810Stop^* heterozygous mutant; Wt, wild-type; M, GeneRuler 1 kb DNA ladder, Thermo Scientific. (**C**) Partial protein sequence alignment (aa 801–862) of murine TRPC7 (Q9WVC5, 862 aa) with other species (http://www.uniprot.org, accessed on 31 August 2021). The amino acid residue in red colour shows the position of the *Trpc7^K810Stop^* mutation; non-homologous amino acids are depicted in grey colour.

Consecutively, exome sequencing using genomic DNA of two phenotypically heterozygous mutant C3H mice (10412812m and 10415366f) was carried out for the search of the causative mutation. Compared to wild-type C3H mice, the analysis resulted in the detection of a single base exchange from A to T leading to the exchange of codon K810 (AAG) to a preliminary stop codon (TAG) in the gene *Trpc7* (transient receptor potential cation channel, subfamily C, member 7) on MMU 13, 56.8 Mb. Therefore, the name of line SMA002 was designated as *Trpc7^K810Stop^*. Allelic differentiation of the *Trpc7^K810Stop^* mutation was performed by PCR-RFLP since the point mutation generated a novel restriction site for the enzyme FspBI (Figure 2). TRPC1-TRPC6 are encoded on different mouse chromosomes.

*Trpc7* encodes a receptor-activated non-selective calcium permeable cation channel. The murine *Trpc7* gene encodes an 862 amino acid protein consisting of an N terminal cytoplasmic domain, six helical transmembrane domains and the cytoplasmic C terminus (aa 673–862). The amino acid sequence of the protein is highly conserved among different species including the C terminal cytoplasmic domain which is affected by the *Trpc7^K810Stop^* mutation (Figure 2, http://www.uniprot.org, accessed on 31 August 2021).

### 3.3. Breeding Trpc7^K810Stop^ Homozygous Mutant Mice

The analysis of the viability of homozygous mutants in this line was already done before the causative mutation was identified. For this purpose, phenotypically heterozygous mutant F1 hybrid mice with the mixed C3H and C57BL/6J genetic background were bred. Due to the debilitating mutant phenotype offspring of two early developmental stages were produced, at embryonic day 14 (which is after the developmental maturity of the placenta) and newborn mice. After the identification of the causative mutation, the tissue samples of the F2 hybrid offspring were re-analyzed for the causative *Trpc7^K810Stop^* mutation.

At embryonic day 14, 91 embryos derived from 11 litters resulted in 33 (36%) wild-type, 42 (46%) heterozygous mutant and 16 (18%) homozygous mutant embryos. The 28 newborn mice analyzed from 4 litters classified in 9 (32%) wild-type, 10 (36%) heterozygous mutant and 9 (32%) homozygous mutant animals. Thus, newborn *Trpc7^K810Stop^* homozygous mutant mice are viable at least on the mixed C3H and C57BL/6J genetic background. Further analyses have to be carried out with older offspring on the C3H and/or C57BL/6J inbred genetic background.

## 4. Discussion

A single base exchange was identified in the ENU mutagenesis-derived dominant mutant mouse line SMA002 leading to the establishment of the mutant mouse line *Trpc7^K810Stop^*. Heterozygous mutants showed the combined appearance of growth deficit and abnormal behaviour.

The causative mutation *Trpc7^K810Stop^* in exon 12 affects the highly conserved region coding for the cytoplasmic C terminus (aa 673-862). Two *Trpc7* knockout mouse lines are published exhibiting the targeted deletion of exon 1 and exon 5 of *Trpc7*, respectively (http://www.informatics.jax.org, accessed on 31 August 2021). Homozygous mutant mice of both lines were described to be viable and to show no obvious differences in weight and size when compared to wild-type littermates [3,4]. In addition, knockout mice lacking the expression of all seven members of the TRPC family (TRPC1-TRPC7) were viable [5].

The spontaneous appearance of two pathological main symptoms in the *Trpc7^K810Stop^* heterozygous mutant mice leads to the assumption of a dominant negative effect of this mutant protein. This confirms observations of differences in ENU mutant versus knockout phenotypes for various genes [15]. Newborn *Trpc7^K810Stop^* homozygous mutant mice indicate that the mutant protein in the homozygous state is compatible with viability at least in the first days post partum.

The abnormal behavioural phenotype found in the mutant line *Trpc7^K810Stop^* may be in line with the detection of a role of *Trpc7* in triggering epileptic seizures by carrying out induction experiments in *Trpc7* knockout mice. The genetic ablation of TRPC7 in a mixed 129/Sv and C57BL/6 genetic background was shown to disrupt acute severe seizures induced by pilocarpine in mice. This disruption was associated with a reduction in pilocarpine-induced increase in gamma wave activity that precedes the acute seizures [6].

The wellbeing of the *Trpc7^K810Stop^* mutant mice was supposed to be altered by the seizure-like attacks itself as well as by secondary consequences, e.g., on the feed and water intake and the wake-sleep-cycle. The growth deficit of the *Trpc7^K810Stop^* mutant mice may be a consequence of the seizure-like attacks particularly observed in the young mice, but may also be caused by the potential role of TRPC7 suggested in cell growth and progression [1]. Older *Trpc7^K810Stop^* mutant mice on the genetic background of C57BL/6J inbred mice inconstantly showed skin alterations of various degrees which may reflect the TRPC7 function described in the skin. Here, TRPC7 was characterized as a nociceptive mechanoreceptor, and it was described to mediate UVB-induced epidermal pathology, epidermal aging, and skin tumour initiation and growth in mice [7]. This might explain the increased self-grooming and scratching behaviour detected in our *Trpc7^K810Stop^* heterozygous mutants.

The debilitating mutant phenotype resulted in cases of *Trpc7^K810Stop^* heterozygous mutants showing increasing burden. Thus, the mutant mice were carefully monitored, and euthanasia was carried out to prevent the appearance of severe adverse effects. In addition, breeding of the mutant line was stopped after successful sperm cryo-preservation to allow rederivation of the line for further defined research projects. Cryo-preserved sperm of the mutant line *Trpc7^K810Stop^* is available upon request for scientific projects.

In humans, TRPC7 mRNA is broadly expressed in the central nervous system as well as in peripheral tissues [1]. As both other TRPC subgroup members TRPC3 and TRPC6, TRPC7 is suggested to play a potential role in the physiology and pathology of neurological disorders [2,16], in the cardiovascular system [9,10,17], and in cancer cell growth and progression [1,7,8].

TRPC proteins assemble as homomeric as well as heteromeric channels, often but not always formed by members of the same subgroup. Thus, channel heteromerization with other TRPC proteins or proteins of the TRP superfamily may enhance the diversity of signaling mechanisms through TRPC7. However, up to now very little is published regarding the status of endogenous heteromeric TRPC channels and their physiological functions [1,18].

In total, in contrast to the published phenotype of *Trpc7* knockout mice, the *Trpc7^K810Stop^* mutation leads to a dominant negative effect of the mutant protein. This was observed in the genetic background of two different inbred strains. An analogous phenotype may occur in other species and/or by the expression of other mutant TRPC7 proteins. However, analysis of the mutant protein as well as the phenotypic analysis of homozygous mutants were not yet carried out in line *Trpc7^K810Stop^*.

## Figures and Tables

**Table 1:** Clinical chemical and hematological analysis of line *Trpc7^K810Stop^*.

Parameter	Heterozygous Mutant Males	Wild-Type Males	Heterozygous Mutant Females	Wild-Type Females
Na (mmol/L)	150 ± 6	152 ± 7	151 ± 8	153 ± 7
K (mmol/L)	4.4 ± 0.2 ^a^	4.6 ± 0.3	4.0 ± 0.4 ^c^	4.4 ± 0.3
Ca (mmol/L)	2.2 ± 0.1	2.2 ± 0.1	2.2 ± 0.1 ^c^	2.3 ± 0.1
Cl (mmol/L)	109 ± 8	109 ± 7	109 ± 10	110 ± 8
P_i_ (mmol/L)	1.8 ± 0.2	2.0 ± 0.3	2.0 ± 0.2	2.1 ± 0.4
Total protein (g/dL)	5.2 ± 0.2 ^c^	5.5 ± 0.2	4.9 ± 0.2 ^c^	5.4 ± 0.2
Creatinine (mg/dL)	0.21 ± 0.05	0.23 ± 0.06	0.20 ± 0.04 ^a^	0.22 ± 0.04
Urea (mg/dL)	49 ± 6	48 ± 7	48 ± 6 ^c^	39 ± 6
Cholesterol (mg/dL)	102 ± 7 ^c^	132 ± 14	85 ± 8 ^c^	107 ± 12
Triglycerides (mg/dL)	75 ± 23 ^c^	145 ± 57	72 ± 14 ^c^	156 ± 59
α-Amylase (U/L)	2936 ± 266 ^b^	3165 ± 272	2675 ± 298	2783 ± 276
ALT (U/L)	11 ± 2	13 ± 7	12 ± 2 ^a^	11 ± 2
AST (U/L)	31 ± 10	27 ± 8	32 ± 6	29 ± 5
CK (U/L)	73 ± 97	57 ± 64	47 ± 25	59 ± 34
AP (U/L)	188 ± 23 ^b^	169 ± 20	226 ± 18	222 ± 22
Uric acid (mg/dL)	2.8 ± 1.7	2.4 ± 1.6	2.3 ± 1.5	1.8 ± 1.4
Glucose (mg/dL)	101 ± 30	112 ± 28	86 ± 23 ^a^	104 ± 29
WBC (10^3^/µL)	5.1 ± 2.4	4.9 ± 2.7	5.7 ± 2.7	4.7 ± 2.1
RBC (10^6^/µL)	9.3 ± 0.5	9.4 ± 0.4	9.1 ± 0.5	9.1 ± 0.5
PLT (10^3^/µL)	506 ± 109	537 ± 98	503 ± 89	500 ± 126
HGB (g/dL)	14.7 ± 0.8	15.0 ± 0.6	14.7 ± 0.8	14.6 ± 0.7
HCT (%)	44.3 ± 2.7	45.1 ± 1.9	43.6 ± 2.3	43.8 ± 2.3
MCV (fL)	47.9 ± 0.8	47.9 ± 0.6	47.7 ± 0.5 ^b^	48.2 ± 0.6
MCH (pg)	15.9 ± 0.3	15.9 ± 0.5	16.1 ± 0.4	16.1 ± 0.3
MCHC (g/dL)	33.3 ± 0.8	33.1 ± 0.9	33.8 ± 0.7	33.5 ± 0.8

Twelve-week-old mice were tested. *n* = 22–25 per sex and genotype. Data are presented as mean ± standard deviation. Significance vs. wild-type controls: a, *p* < 0.05; b, *p* < 0.01; c, *p* < 0.001. Creatinine, plasma creatinine analyzed by the Jaffé method; ALT, alanine aminotransferase (EC 2.6.1.2); AST, aspartate aminotransferase (EC 2.6.1.1); AP, alkaline phosphatase (EC 3.1.3.1); CK, creatine kinase (EC 2.7.3.2). WBC, white blood cell count; RBC, red blood cell count; PLT, platelet count; HGB, hemoglobin; HCT, hematocrit; MCV, mean corpuscular volume; MCH, mean corpuscular hemoglobin; MCHC, mean corpuscular hemoglobin concentration.

## Data Availability

The data presented in this study are available within the article.
